# Pathogenic SCN5A Mutation and Thyrotoxicosis-Related Neurological Syndrome: Casual or Causal Relationship?

**DOI:** 10.3390/brainsci13071049

**Published:** 2023-07-10

**Authors:** Yangqi Xu, Lin Zhao, Jihong Dong, Jingjing Jiang, Lirong Jin

**Affiliations:** 1 Department of Neurology, Zhongshan Hospital, Fudan University, Shanghai 200030, China; yangqixu666@163.com (Y.X.); dong.jihong@zs-hospital.sh.cn (J.D.); 2 Department of Endocrinology, Zhongshan Hospital, Fudan University, Shanghai 200030, China; zhao.lin@zs-hospital.sh.cn (L.Z.); jiang.jingjing@zs-hospital.sh.cn (J.J.)

**Keywords:** hyperthyroidism, myalgia, stiffness, myoclonus, SCN5A

## Abstract

Background: Various neurologic complications of hyperthyroidism are reported, and most of these complications are reversible with the amelioration of thyrotoxicosis. We report a previously undescribed concurrence of hyperthyroid-associated exercise-induced myalgia and stiffness, pyramidal tract dysfunction, and myoclonic movements that make an initial clinical diagnosis difficult. Case presentation: A 17-year-old male was hospitalized in the department of neurology, presenting with a 4-year history of severe exercise-induced myalgia and stiffness, weakness of lower limbs, and myoclonic movements. Laboratory investigations unexpectedly revealed hyperthyroidism. MRI of the brain and spine, electrophysiology, and whole exome sequencing were also performed. Antithyroid therapy led to marked improvement of neurologic symptoms, accompanied by a significant improvement of the time-dependent decline in compound muscle action potentials (CMAP) amplitudes after exercise and normalization of the prolonged QTc interval. Genetic analysis identified a rare variant in SCN5A. Conclusion: This case report provides important insights into the relationship between hyperthyroidism and neurologic/cardiac complications, particularly in those with a genetic predisposition. SCN5A mutation possibly plays a role in the complex neurological syndrome associated with hyperthyroidism. Further studies are warranted to better understand the underlying mechanisms and potential therapeutic options for these complex conditions.

## 1. Introduction

Hyperthyroidism has been well known to disrupt the neurologic system, though most of the neurologic symptoms associated with thyrotoxicosis are reversible with antithyroid therapy [1,2,3,4]. Hyperthyroidism can also contribute to arrhythmia, particularly atrial fibrillation. Here, we report a 17-year-old juvenile presenting with exercise-induced myalgia and stiffness of lower limbs, leg weakness, and myoclonic jerks of quadriceps muscles. Whole exome sequencing was performed, demonstrating a heterozygous pathogenic missense variant in the SCN5A (c.4018G>A, p.V1340I). The symptoms and signs were improved dramatically within two months of antithyroid therapy. In this case, we summarized the clinical features caused by hyperthyroidism and explored the underlying mechanisms according to the previous literature.

## 2. Case Presentation

**Figure 1:**
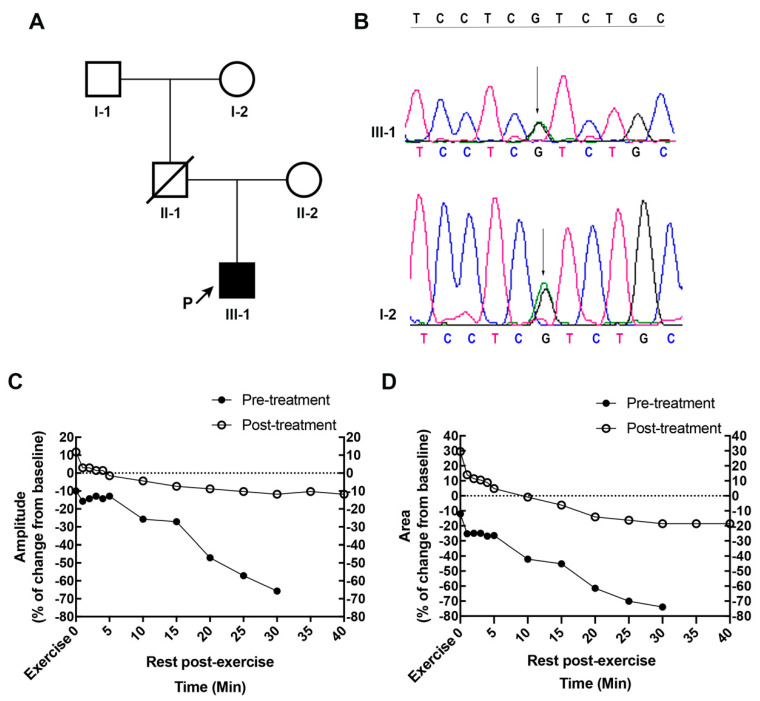
(**A**) Pedigree structure of patient’s family. Circles indicate females; squares indicate males. Symbols with a crossed line indicate deceased individuals. The proband is marked by an arrow and the letter “P”. (**B**) The patient’s whole exome sequencing demonstrated c.4018G>A variation in the SCN5A gene. The same variation was found in his grandmother, confirmed by Sanger sequencing. The exercise test of post-treatment showed a great increase in CMAP amplitude (**C**) and area (**D**) immediately after 5 min of exercise and a smaller decrease rate after exercise when compared with the pre-treatment. The amplitude and area of CMAP expressed as a percentage of change from baseline.

The patient started to present with severe myalgia, weakness of legs, and tremor of hands during high school training when he was 13 years old. Those symptoms gradually eased after rest for half a month. Later, exercise-induced myalgia frequently led to falls, and his symptoms relapsed. At the age of 16, he developed dysphagia, and he was confined to bed for months due to the stiffness and myalgia of legs. He had difficulty bending his knees and could not ambulate independently due to his leg spasticity (Supplementary videotape segment 2). A detailed investigation of family history revealed that the patient’s father died of cardiac arrest in his thirties, and his grandmother also had a history of hyperthyroidism in her thirties (Figure 1A).

Upon admission at age 17, he looked very thin and weighed 60 Kg, and his height was 1.8 m. His palms were abnormally over-sweating which was associated with palmar hyperhidrosis. No exophthalmos and palpable thyroid enlargement were found. Mental function and cranial nerves were normal. A fine intention tremor of bilateral hands and noticeably increased tone of his legs were present. Involuntary jerks without pain were noted in the thigh muscles of the right leg and became more obvious with exertion (Supplementary videotape segment 1). Tendon reflexes were exaggerated on both sides. Abdominal reflexes were brisk. Moderate (grade 3 to 5) weakness of muscular groups of lower limbs was evident, most prominent proximally. Finger flexion reflex and extensor plantar response could not be elicited bilaterally.

Laboratory tests revealed undetectable serum thyroid stimulating hormone (TSH) with raised free T3, free T4, and TSH receptor antibodies (TRAb), suggesting hyperthyroidism (Table 1). Blood cell count, electrolyte, glucose, liver function, creatine kinase (CK), rheumatic antibodies, folate, and vitamin B12 were all within normal range.

The patient’s electrocardiogram (ECG) showed sinus tachycardia of 119 beats per minute with a prolonged QTc of 453 ms (normal range: 350–440 ms). An ultrasound of the thyroid gland showed an enlarged gland with increased parenchymal vascularity, the right lobe of 20 mm × 23 mm × 51 mm and left lobe of 18 mm × 19 mm × 50 mm, and isthmus of 3.0 mm. Pharyngolaryngoscopic study revealed neither an organic lesion nor obstruction, and normal vocal movement. Detailed magnetic resonance imaging (MRI) of the brain and cervical and thoracic spinal cord were unremarkable. Needle electromyography (EMG), nerve conduction studies (NCS), and electroencephalographic studies were within normal limits. In long exercise test (LET), a decrement of the compound muscle action potentials (CMAP) amplitude and area immediately after exercise followed by a significant time-dependent gradual decline was observed up to 30 min postexercise (Figure 1C,D). Genetic testing was performed for the patient’s family history and revealed a heterozygous missense mutation of the SCN5A gene (NM_198056: c.4018G>A) resulting in the substitution of isoleucine for valine at codon 1340 (p.V1340I) in the patient. The same variant was also identified in his grandmother, implying that the patient’s father also carried the variant (Figure 1B). The SCN5A gene encodes the major cardiac voltage- gated sodium channel α-subunit Nav1.5, and the SCN5A V1340I variant has been previously reported to be detrimental in patients with Brugada syndrome (BrS) [5]. In this case, the cardiac arrest of the patient’s father was also highly suggestive of the pathogenicity of the variant. This variant has not been reported in 1000 genomes and has an allele frequency of 0.0003 in East Asian people in the ExAC database. In silico predictions (SIFT, MutationTaster2, and PolyPhen-2) suggested its pathogenicity. We interpreted the variant to be pathogenic according to the American College of Medical Genetics and Genomics (ACMG) standards and guidelines.

The patient was diagnosed with Graves’ disease, and antithyroid therapy with methimazole and beta-blockers was initiated. Within two weeks, his muscle hypertonia of lower limbs eased dramatically, and his gait returned to normal (Supplementary videotape segment 3). By two months, his body weight increased to 65 Kg. Dysphagia and myoclonic movements were no longer present. Exercise-induced myalgia and stiffness occurred only if he kept walking. Extremity strength and hyperreflexia remained almost unchanged. His ECG reverted to normal sinus rhythm of 77 beats per minute with a QTc of 400 ms. LET showed an increase in CMAP amplitude and area immediately after exercise and a slight decline postexercise. The greatest decrease in CMAP amplitude at 30 min postexercise was greatly improved from 65.7% of the baseline value of pre-treatment to 10.3% of post-treatment.

## 3. Discussion and Conclusions

The patient in this case presented with a complex array of symptoms, including exercise-induced myalgia and spasticity, weakness of the lower limbs, and involuntary jerks in the thigh muscles, which made the diagnosis difficult, particularly due to the concurrent hyperthyroidism. Notably, while hyperreflexia and muscle weakness persisted, other symptoms remitted dramatically after antithyroid treatment, suggesting that hyperthyroidism contributed to the patient’s neurologic deficit.

Evidence for pyramidal tract dysfunction in our case was manifested by symptoms consisting of increased tendon reflexes in all four limbs and weakness of the lower limbs. Based on the presenting literature, the underlying etiology of dysphagia in patients with thyrotoxicosis includes bulbar or esophageal myopathy, electrolyte abnormalities like hypokalemia, and mechanical cause by enlarged goiter [6,7,8]. The EMG and pharyngolaryngoscopy results of the patient could not provide sufficient evidence for thyrotoxic bulbar myopathy. We speculate that dysphagia in this patient is possibly due to the thyroid-related corticobulbar disfunction. The myoclonic movements in the thigh muscles were possibly of spinal origin for the muscles controlled by one or a few contiguous segments of the spinal cord and the pattern of jerks [9,10]. We summarized the clinical features in the reported cases of myoclonus with hyperthyroidism (Table 2). The types of myoclonus in relationship to hyperthyroidism include platysmal myoclonus, generalized myoclonus, and the other unclassified myoclonus [11]. The underlying mechanisms involved might be the autoimmunity or the decreased neuronal firing threshold associated with hyperthyroid state. Dysphagia and the myoclonic movements were resolved greatly following the restoration of euthyroidism, whereas the signs of extremity strength and hyperreflexia improved slowly.

Myalgia is a rare symptom of hyperthyroidism. In one case report, myalgia associated with stiffness was described in a 50-year-old female patient with Graves’ disease, which resolved completely within one month of antithyroid therapy [2]. In the present case, the patient experienced exercise-induced myalgia and exercise intolerance, which was also improved in parallel with the restoration of euthyroidism. The myalgia with electrodiagnosis showing no myopathic features and normal serum CK implied a functional or other muscle disorder rather than myopathies [12].

The exercise tests (ET) are often used to diagnose skeleton muscular ion channelopathies and could reflect the muscle membrane excitability [13]. An immediate decrease in CMAP amplitudes just after exercise and a great time-dependent decline in CMAP amplitudes postexercise suggest fiber inexcitability in the muscle membrane of the patient. The great improvement in the exercise test of post-treatment provided objective evidence of muscle membrane excitability abilities in line with the patient’s euthyroid state. Thyroid hormone (TH) directly and indirectly stimulates the Na/K ATPase pump and eventually contributes to hyperpolarization of the muscle cell membranes [14]. Myalgia and stiffness could be atypical phenotype of skeletal muscle channelopathies and lead to a positive LET results presenting as a post-exercise decrement of more than 40% from peak CMAP amplitude [15,16]. Thus, we infer that exercise-induced myalgia and muscle stiffness in this patient possibly associate with the function of ion channel which is sensitive to excess TH. Importantly, a preexisting latent abnormal excitability of the muscle membrane could be essential for this process and contribute to the occurrence and development of these symptoms [17]. Whether the SCN5A mutation participated in increasing the susceptibilities of abnormal muscle membrane excitability in this patient remains unknown and requires further investigation.

The mechanism underlying the neurological complications of hyperthyroidism remains unknown. Thyroid hormone receptors are widely distributed throughout the body, including the central nervous system and skeletal muscles [18,19]. Hyperthyroidism selectively increases the oxidative metabolism of slow-oxidative motor units, leading to enhanced oxidative phosphorylation and increased expression of uncoupling proteins in muscle mitochondria [20,21]. This can result in a loss of functioning motor units, likely due to the overstimulation of motoneuron metabolism in patients with thyrotoxicosis [22]. Additionally, TH can reduce the number of choline acetyltransferase (ChAT)-immunoreactive neurons in the lumbar tract of the spinal cord by regulating gene transcription in hyperthyroid rats [23]. Furthermore, in hyperthyroid rats, the amplitude of miniature end-plate potentials and muscle-membrane potential were significantly reduced, and some muscle fibers were inexcitable even to direct stimulation [24].

TH has profound influences on the neurologic as well as cardiovascular systems. In patients with a genetic predisposition for arrhythmias, thyrotoxicosis can lower the arrhythmia threshold, and in rare cases, ventricular fibrillation may occur. A recent case report described a patient carrying the SCN5A mutation who developed ventricular fibrillation in the setting of hyperthyroidism [25]. SCN5A mutations have been implicated in the pathogenesis of long QT syndrome, BrS, and cardiomyopathy [26]. The impact of thyrotoxicosis on the probability of lethal arrhythmia in those carriers of pathogenic SCN5A mutation remains to be investigated.

Nav1.5, the pore forming α-subunit of the voltage-dependent cardiac Na+ channel, is not exclusively expressed in heart but has also been detected in the brain and gastrointestinal smooth muscle [27]. It has been reported that SCN5A-mediated abnormal cardiac phenotypes overlap with epilepsy [28,29,30], hyperthyroidism [25], and irritable bowel syndrome. Thus, some researchers propose concepts of overlap syndromes and systemic disorders in relation to SCN5A-mediated cardiac disfunctions [31]. However, the impact of SCN5A variants on neuromuscular system has not been reported. In skeletal muscle, Nav1.5 is the dominant isoform during embryonic myogenesis, and it is gradually replaced by the adult isoform, Nav1.4 encoded by SCN4A. Nav1.5 re-appears in adult skeletal muscle following denervation, and participates in the generation of fibrillation potentials in denervated skeletal muscle [32,33]. More studies are required to elucidate the potential relationship between SCN5A and its overlap syndromes such as thyroid hormone imbalances and/or neurologic disfunctions.

This case expands the spectrum of chronic thyrotoxicosis-induced disorders. Exercise-induced myalgia and stiffness could resolve rapidly after the restoration of euthyroidism and possibly associate with abnormal muscle membrane excitability. This case also underscores the need for vigilant monitoring of potential cardiac complications in patients with chronic thyrotoxicosis, highlighting the complex associations among the endocrine, neurologic, and cardiovascular systems linked by TH.

**Table 2:** The reported cases of myoclonus due to hyperthyroidism.

Study, Year	Age, Sex	Thyroid Function	Myoclonus	Other Symptoms	Underlying Mechanism
Teoh et al., 2005 [4]	Young, female	Subclinical hyperthyroidism	Platysmal Myoclonus	None	NA
Liao et al., 1993 [34]	52 y, female	Hyperthyroidism and thymoma	Myoclonus	None	Autoimmunity
Kuwahara et al., 2013 [35]	29 y, female	Graves’ ophthalmopathy, TSAb positive, normal fT3 and fT4	Generalized myoclonus	Oscillopsia, truncal ataxia	Autoimmunity
Loh et al., 2005 [36]	40 y, male	Graves’ disease	Spasmodic truncal jerking	Proximal myopathy	Hyperthyroid state decreased the neuronal firing threshold

T4, Thyroxine; T3, Triiodothyronine; NA, not available; TSAb, Thyroid stimulating antibodies.

## Figures and Tables

**Table 1:** The thyroid function test of the patient.

Lab Values	Pre-Treatment	Two Weeks Post-Treatment	Two Months Post-Treatment	Reference Range
Free T4 (pmol/L)	>100	44.6	20.6	12.0–22.0
Free T3 (pmol/L)	37.1	20.4	8.6	3.1–6.8
TSH (μIU/mL)	<0.005	<0.005	<0.005	0.27–4.2
TRAb (IU/L)	8.5	8.4	4.3	<1.75
TSAb (UI/L)	22.2	/	/	<0.55
TPOAb (IU/mL)	9.6	/	/	<34.0

T4, Thyroxine; T3, Triiodothyronine; TSH, Thyroid stimulating hormone; TRAb, TSH-receptor antibodies; TSAb, Thyroid stimulating antibodies; TPOAb, Thyroid peroxidase antibodies.

## Data Availability

The data presented in this study are available on request from the corresponding author.

## Supplementary Materials

The following are available online at https://www.mdpi.com/article/10.3390/brainsci13071049/s1. Supplementary video legend: Segment 1. Patient at rest presented spontaneous myoclonus in the right quadriceps. Muscle exertion worsened the severity of myoclonus. Segment 2. Patient had difficulty to ambulate for the spasticity of the lower limbs. Segment 3. Patient had great improvement of walking after treatment, with the exception of weakness of the lower limbs.
